# Folliculitis caused by *Pantoea dispersa* as a souvenir from a self-discovery excursion in bat caves

**DOI:** 10.1016/j.jdcr.2022.05.009

**Published:** 2022-05-18

**Authors:** Sarah Preis, Kathrin Schröder, Tilo Biedermann, Alexander Zink

**Affiliations:** a Department of Dermatology and Allergy, School of Medicine, Technical University of Munich, Munich, Germany; b Institute for Medical Microbiology, Immunology and Hygiene, School of Medicine, Technical University of Munich, Munich, Germany; c Division of Dermatology and Venereology, Department of Medicine Solna, Karolinska Institutet, Stockholm, Sweden

**Keywords:** folliculitis, *Pantoea dispersa*, skin infection, *P dispersa*, *Pantoea dispersa*

## Introduction

Increasing globalization and access to exotic travel destinations have led to a change in the spectrum of diseases. Recent disease outbreaks, such as the COVID-19 pandemic, have shed light on animal pathogens turning into human health threats. Typical examples in dermatology include *larva migrans*, but relatively unknown bacterial infections are gaining ground and challenging clinicians. Compared with animal pathogens, plant pathogens are exceptionally rare pathogens encountered in clinical settings with until now unclear underlying pathogenetic mechanisms.

Herein, we report the case of a generalized skin infection caused by the plant pathogen *Pantoea dispersa* potentially imported from a cave trip in Latin America.

## Case report

**Fig 1:**
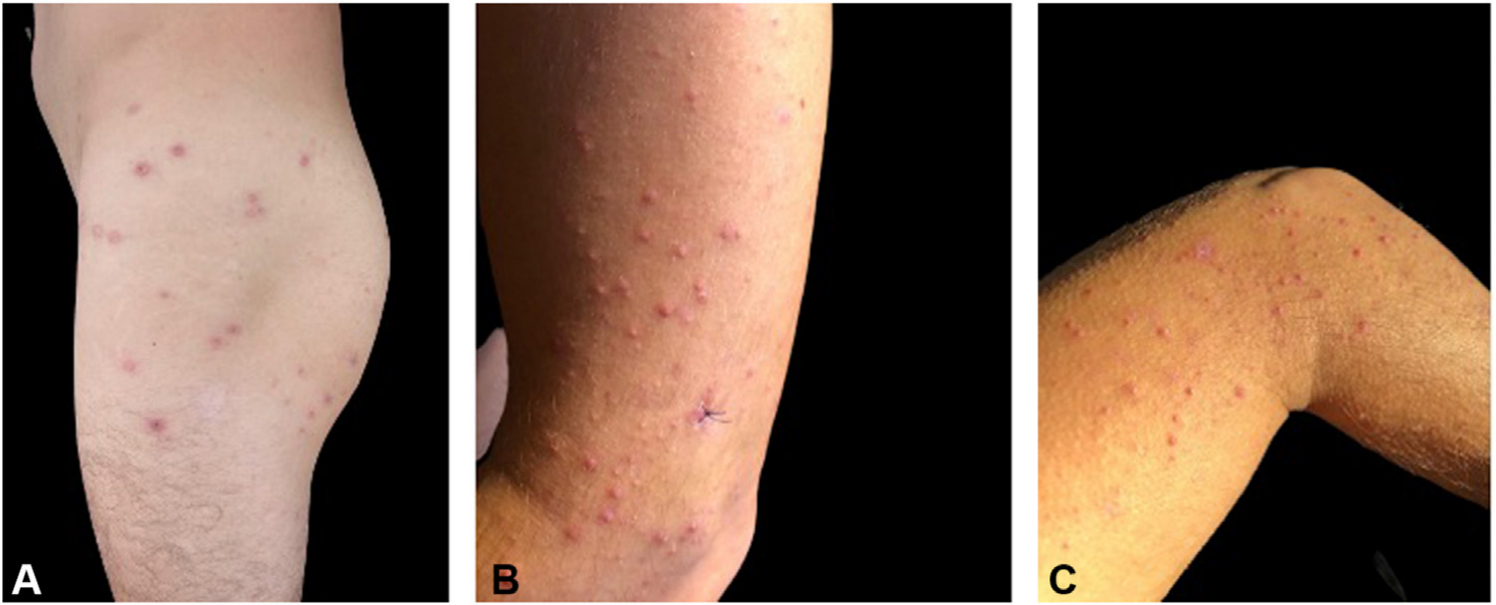
**A-C**. Physical examination at initial presentation revealed erythematous, slightly pruritic papules next to disseminated pustules around inflamed hair follicles alongside small, shallow, punched-out ulcers with thick, red-brown crusts and surrounding erythema on the whole integument.

**Fig 2:**
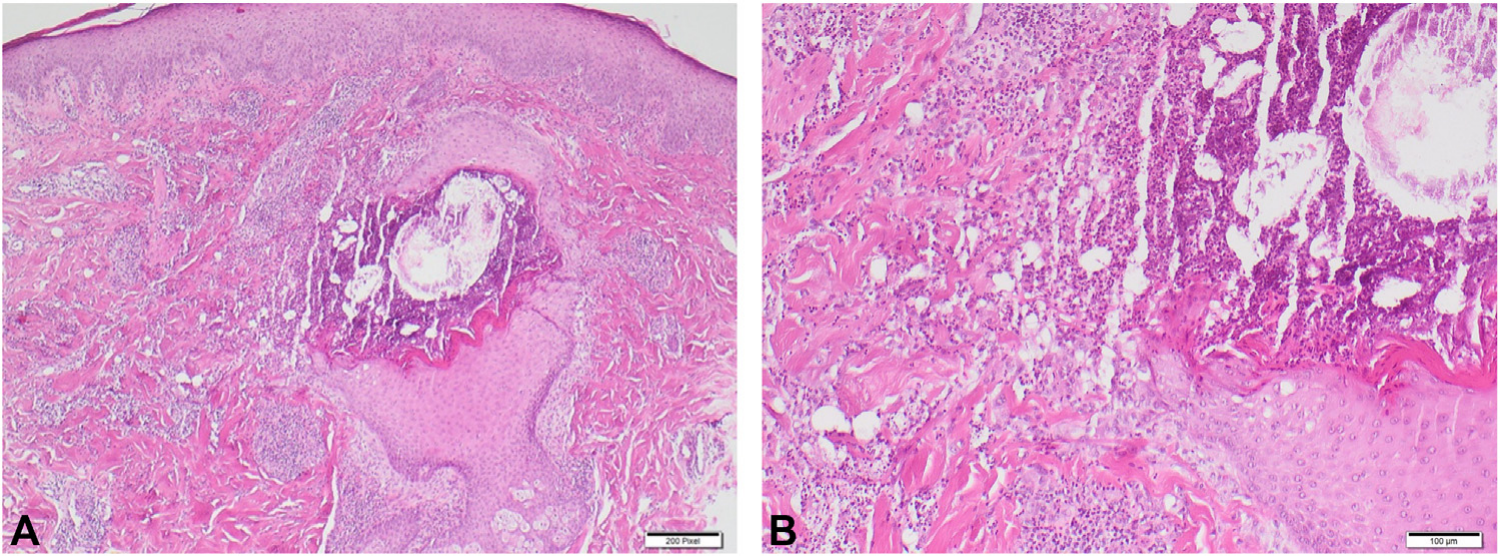
**A** and **B.** Biopsies of the trunk and the arm revealed an abscessing folliculitis and perifolliculitis. (**A** and **B**, Hematoxylin-eosin stain; original magnification: **A**, ×40 and **B**, ×100).

A 33-year-old man presented to our outpatient department after returning from a trip to the Mexican jungle. He reported having spent 6 hours naked in a dark, humid cave inhabited by bats to find his inner balance. Four days after returning to society, he and his fellow meditative companions developed numerous yellow-reddish pustules, first occurring on body parts that were in contact with the soil of the cave. Under the assumption that the condition was a vasculitis, the patient was treated with systemic corticosteroids (40 mg/d for 7 days) by a local general practitioner, which unfortunately aggravated the symptoms and the skin lesions. At initial presentation, physical examination revealed erythematous, slightly pruritic papules next to disseminated pustules around inflamed hair follicles alongside small, shallow, punched-out ulcers with thick, red-brown crusts and surrounding erythema on the entire integument (Fig 1, *A-C*). Biopsies of the trunk and the arm were performed, showing an abscessing folliculitis and perifolliculitis (Fig 2, *A* and *B*). No organisms were present in the biopsies. Blood testing revealed a slightly increased leukocytosis. An HIV test was negative. A microbiologic culture of the pustules revealed *P dispersa*. We thereby diagnosed a *P dispersa* associated folliculitis. In accordance with the antibiogram, the patient was prescribed trimethoprim-sulfamethoxazole (960 mg twice daily) for 7 days and an antiseptic washing lotion, which led to an abatement of the skin lesions within 10 days.

## Discussion

Folliculitis affects the superficial aspect of the hair follicle and can involve the perifollicular area.^1^ Folliculitis has a broad clinical presentation because of its etiology, but it mostly manifests in acute cases as 1-mm–wide pustules and papulopustules, with the possibility of developing into nonhealing ulcerations with crusts.^1^ Common bacteria that cause folliculitis are *Staphylococcous aureus* and species of *Streptococcus*, *Pseudomonas*, and *Proteus*.^2^ Folliculitis caused by gram-negative bacteria is rare,^2^ and to our knowledge *Pantoea* species as a cause of folliculitis have not been described in the literature until now.

*Pantoea* is a gram-negative, versatile member of the Enterobacteriaceae family that can be isolated from a variety of environments.^3^ Macroscopically it presents as a yellow-pigmented, rod-shaped, motile bacterium.^3^ The very first identified members were observed to cause wilting, soft rot, and necrosis in a variety of agriculturally relevant plants, such as beets, maize, and epiphytes. *Pantoea* species have since been isolated from many aquatic and terrestrial environments and described to have an association with insects and other animals.^3^ Furthermore, recent research has disclosed data on the bioremediation potential of *Pantoea* species and on its role as a immunopotentiator in the development of supportive drugs that treat melanoma, infections, allergy, and immunosuppression.^4^ These mechanisms can primarily be explained by the activation of macrophages by lipopolysaccharides derived from *Pantoea* species, leading to activation of the immune system and thereby antiinflammatory effects.^4^ The genus *Pantoea* is divided into 20 different species and includes *Pantoea agglomerans*, *P septica*, *P ananatis,* and *P dispersa.*^3^ *P dispersa* inhabits plants, soil, and humid ground.^5^ Dismissed in the past as a plant pathogen that forms host associations with different plants and fungi, recent evidence suggests an additional role in human disease.^3^ *P agglomerans*, the most common member, causes opportunistic human infections; eg, wound infections, after contact with plant material in addition to hospital-acquired infections.^4^ Although in most cases of generalized folliculitis amoxicillin is used as treatment, *Pantoea* species are often resistant to common clinically used antibiotics, such as penicillin G, bacitracin, rifampicin, vancomycin, and fosfomycin.^6^

The literature reports of only 6 cases of human infections caused by *P dispersa*. The clinical presentation of *P dispersa* infections in these cases varied from bacteremia to respiratory tract infections. Two patients were neonates, who developed early-onset sepsis. Treating the newborns with meropenem and amikacin according to an antibiogram and a resistogram improved symptoms.^7^ In the first report of an adult infected with *P dispersa*, a 71-year-old immunocompromised woman with acute myeloid leukemia and multiple myeloma developed a respiratory tract infection that was treated successfully with test-appropriate antibiotic therapy.^8^ Furthermore, the literature contains 3 reports on bloodstream infections caused by *P dispersa* in a 64-year-old man after implantation of a pacemaker, a 38-year-old woman with acute cholangitis, and a 23-year-old woman with lethal sepsis.^9^ Although to our knowledge no cases of skin infections with *P dispersa* have been described, the literature reports cases of skin infections with *P agglomerans*.^4^ These include a 58-year-old woman presenting with a wound infection after a penetrating plant injury and a patient with multiple skin eruptions presenting as small papules.^10^ Overall, *P dispersa* infections can apparently occur in immunocompetent patients in the same manner as in immunocompromised hosts, although the literature on this is very limited.

Detecting *P dispersa* remains challenging because of its rarity and because of difficulty in correct identification. As *P dispersa* belongs to the Enterobacter species, some cases may be incorrectly identified as being caused by species of Enterobacteriaceae.^9^ Common diagnostic tools, such as the MALDI Biotyper often misidentify Erwiniacea as *Klebsiella* species.^9^ A detailed anamnesis can be helpful in these vague cases. All species of *Pantoea* can be isolated form plant, soil, and feculent material.^3^ As our patient spent hours in a humid cave inhabited by bats and other animals, the existing fecal matter and moist soil may have provided an ideal breeding ground for *Pantoea*.^3^ It stands to reason that he contracted this rare infection during his trip to the jungle.

The pathogenetic mechanisms of *P dispersa* remain unclear, and the current case report generates a range of questions as to what degree these plant pathogens can cause infections in humans.^9^

## Conflicts of interest

None disclosed.

## References

[1] Luelmo-Aguilar J., Santandreu M.S. (2004). Folliculitis: recognition and management. Am J Clin Dermatol.

[2] Chiller K., Selkin B.A., Murakawa G.J. (2001). Skin microflora and bacterial infections of the skin. J Investig Dermatol Symp Proc.

[3] Walterson A.M., Stavrinides J. (2015). *Pantoea:* insights into a highly versatile and diverse genus within the Enterobacteriaceae. FEMS Microbiol Rev.

[4] Dutkiewicz J., Mackiewicz B., Kinga Lemieszek M., Golec M., Milanowski J. (2016). *Pantoea agglomerans:* a mysterious bacterium of evil and good. Part III. Deleterious effects: infections of humans, animals and plants. Ann Agric Environ Med.

[5] Brady C., Cleenwerck I., Venter S., Vancanneyt M., Swings J., Coutinho T. (2008). Phylogeny and identification of *Pantoea* species associated with plants, humans and the natural environment based on multilocus sequence analysis (MLSA). Syst Appl Microbiol.

[6] Guevarra R.B., Magez S., Peeters E., Chung M.S., Kim K.H., Radwanska M. (2021). Comprehensive genomic analysis reveals virulence factors and antibiotic resistance genes in *Pantoea agglomerans* KM1, a potential opportunistic pathogen. PLoS One.

[7] Mehar V., Yadav D., Sanghvi J., Gupta N., Singh K. (2013). *Pantoea dispersa:* an unusual cause of neonatal sepsis. Braz J Infect Dis.

[8] Schmid H., Schubert S., Weber C., Bogner J.R. (2003). Isolation of a *Pantoea dispersa*-like strain from a 71-year-old woman with acute myeloid leukemia and multiple myeloma. Infection.

[9] Panditrao M., Panditrao M. (2018). *Pantoea dispersa:* is it the next emerging “monster” in our intensive care units? A case report and review of literature. Anesth Essays Res.

[10] Alpiste F.O., Ezquerra G.M., Pujol R.M. (2021). Wound infection by *Pantoea agglomerans* after penetrating plant injury. Indian J Dermatol Venereol Leprol.

